# The complex effects of the slow-releasing hydrogen sulfide donor GYY4137 in a model of acute joint inflammation and in human cartilage cells

**DOI:** 10.1111/jcmm.12016

**Published:** 2013-01-28

**Authors:** Ling Li, Bridget Fox, Julie Keeble, Manuel Salto-Tellez, Paul G Winyard, Mark E Wood, Philip K Moore, Matthew Whiteman

**Affiliations:** a Pharmaceutical Science Research Division, King's College LondonLondon, England; b University of Exeter Medical School, St. Luke's CampusExeter, Devon, England; c Department of Pharmacology, National University of SingaporeSingapore, Singapore; d Centre for Cancer Research and Cell Biology, Queen's University BelfastBelfast, Northern Ireland, UK; e Synthetic Chemistry Facility, Department of Biosciences, College of Life and Environmental Sciences, University of ExeterExeter, Devon, England

**Keywords:** hydrogen sulfide, GYY4137, inflammation, synoviocyte, cytokines, myeloperoxidase, lipopolysaccharide, Freund's adjuvant, COX-2, TNF-alpha converting enzyme

## Abstract

The role of hydrogen sulfide (H_2_S) in inflammation remains unclear with both pro- and anti-inflammatory actions of this gas described. We have now assessed the effect of GYY4137 (a slow-releasing H_2_S donor) on lipopolysaccharide (LPS)-evoked release of inflammatory mediators from human synoviocytes (HFLS) and articular chondrocytes (HAC) *in vitro*. We have also examined the effect of GYY4137 in a complete Freund's adjuvant (CFA) model of acute joint inflammation in the mouse. GYY4137 (0.1–0.5 mM) decreased LPS-induced production of nitrite (NO_2_^−^), PGE_2_, TNF-α and IL-6 from HFLS and HAC, reduced the levels and catalytic activity of inducible nitric oxide synthase (iNOS) and cyclooxygenase-2 (COX-2) and reduced LPS-induced NF-κB activation *in vitro*. Using recombinant human enzymes, GYY4137 inhibited the activity of COX-2, iNOS and TNF-α converting enzyme (TACE). In the CFA-treated mouse, GYY4137 (50 mg/kg, i.p.) injected 1 hr prior to CFA increased knee joint swelling while an anti-inflammatory effect, as demonstrated by reduced synovial fluid myeloperoxidase (MPO) and *N*-acetyl-β-D-glucosaminidase (NAG) activity and decreased TNF-α, IL-1β, IL-6 and IL-8 concentration, was apparent when GYY4137 was injected 6 hrs after CFA. GYY4137 was also anti-inflammatory when given 18 hrs after CFA. Thus, although GYY4137 consistently reduced the generation of pro-inflammatory mediators from human joint cells *in vitro*, its effect on acute joint inflammation *in vivo* depended on the timing of administration.

## Introduction

Hydrogen sulfide (H_2_S) is produced in mammalian tissues from the amino acids cysteine and homocysteine by the pyridoxal-5′-phosphate (PLP)-dependent enzymes, cystathionine-γ-lyase (CSE; E.C. 4.4.1.1), cystathionine-β-synthetase (CBS; E.C. 4.2.1.22) and 3-mercaptopyruvate sulfurtransferase (3-MST; EC 2.8.1.2; reviewed in [1, 2]). Despite extensive study, the part played by H_2_S in inflammation remains unclear [3]. We demonstrated some years ago that parenteral injection of the ‘fast-releasing’ H_2_S donor, sodium hydrosulfide (NaSH), caused tissue inflammation in the mouse as evidenced by increased organ myeloperoxidase (MPO) activity following tissue neutrophil infiltration and the associated rise in plasma levels of pro-inflammatory cytokines such as TNF-α [4]. In addition, NaSH incresed leucocyte rolling/adhesion in the mesenteric microcirculation as well as neutrophil migration in mice with sepsis induced by caecal ligation and puncture [5]. Moreover, plasma H_2_S concentrations, tissue H_2_S synthesizing activity and CSE expression were all elevated in a number of animal models of inflammation (*e.g*. endotoxic, septic and haemorrhagic shock, pancreatitis, carrageenan-evoked hindpaw oedema; [4, 6–8]) which together are strongly suggestive of a pro-inflammatory role for H_2_S.

However, it is possible that the elevation of vascular and tissue production of H_2_S observed in animal models of inflammation may equally reflect an endogenous attempt to overcome, control or resolve inflammation rather than potentiate or drive the inflammatory response [1, 3]. More recent studies have shown that, depending on the experimental conditions, H_2_S can also exert prominent anti-inflammatory effects [3, 9]. For example, an H_2_S-releasing derivative of diclofenac (*c.f*. diclofenac alone) exhibited enhanced anti-inflammatory activity in endotoxic shock and against carrageenan-induced hindpaw swelling [10, 11]. The slow-releasing, water-soluble H_2_S donor, GYY4137, has also been shown to be anti-inflammatory in a mouse model of endotoxic shock [12] by mechanisms which include reducing macrophage generation of pro-inflammatory mediators such as nitric oxide (^•^NO) and PGE_2_ [12, 13] and the promotion of phagocytosis [14]. GYY4137 also inhibited IL-8 secretion and cell proliferation in primary human airway smooth muscle cells [15] and stimulated the synthesis of the anti-inflammatory chemokine IL-10 in rat plasma during sepsis [12], albeit through undefined signalling pathways. H_2_S has also been reported to promote ulcer healing [16], reduce lung injury due to smoke inhalation [17] and decrease carrageenan-induced hindpaw oedema [18] in the rat. Moreover, NaSH reduced leucocyte infiltration in an air pouch model [18], and is cardioprotective in pigs subjected to ischaemia reperfusion, most likely by reducing formation of pro-inflammatory cytokines such as TNF-α, IL-6 and IL-8 [19]. Thus, the literature is replete with conflicting evidence indicating that H_2_S can be both pro- and anti-inflammatory [1, 3].

There have been few published reports of the role of H_2_S in acute joint inflammation. Human cartilage cells are capable of synthesizing H_2_S as part of an acute response to pro-inflammatory mediators such as TNF-α, IL-6, IL-1β and bacterial lipopolysaccharide (LPS) and cytokine-induced H_2_S synthesis has been proposed as a mechanism to protect joint cells from oxidative injury [20]. Synovial fluid (SF) aspirated from the knee joints of patients with inflammatory joint diseases in reactive [21, 22] and psoriatic arthritides [3] contained higher concentrations of H_2_S than paired plasma samples of SF from age-matched patients with osteoarthritis. Using an animal model of acute monoarthritis *viz*. intra-articular injection of kaolin/carrageenan into the knee joint of mice [23], intra-articular sodium sulfide (Na_2_S) administration caused a dose-dependent reduction in synovial leucocyte adherence and an increase in leucocyte velocity, indicative of an anti-inflammatory effect. We report here studies to probe the effect of H_2_S on inflammatory mediator formation in cultured synoviocytes *in vitro* and in an animal model of acute joint inflammation *in vivo* using a slow-releasing H_2_S donor, GYY4137.

## Materials and Methods

### Reagents

GYY4137 was synthesized in-house as described in [13, 24]. PPM-18 and NS-398 were purchased from Calbiochem Ltd. (Merck, Feltham, UK). The following were purchased from R&D Systems Inc. (Minneapolis, USA): ELISAs for COX-2, iNOS, TNF-α and IL-6, human recombinant tumour necrosis factor–alpha-converting enzyme (TACE) and fluorogenic TACE substrate Mca-Pro-Leu-Ala-Gln-Ala-Val-DPA-Arg-Ser-Ser-Ser-Arg-NH_2_. PGE_2_ ELISA, COX-2 and NOS Activity Assay kits were purchased from Caymen Chemicals (Ann Arbor, MI, USA). The NF-κB activation ELISA was purchased from Active Motif (Carlsbad, CA, USA). Arginine L-[14C(U)] (#NEC267E050UC) was purchased from Perkin Elmer (Cambridgeshire, England). Human recombinant TNF-α, IFN-γ and IL-1β were purchased from PeproTech (Rocky Hill, NJ, USA). Rabbit monoclonal antibodies to IκB-α and phosphor-ÍκB-α (Ser^32^) were purchased from Cell Signalling Technology (Danvers, MA, USA). Rabbit polyclonal antibodies to TACE and TACE/ADAM17 Activation Site were purchased from Santa Cruz Biotechnology (Santa Cruz, CA, USA) and Abcam (Cambridge, England) respectively. All other chemicals and kits including lipopolysaccharide (LPS; *E. coli* 0127:B8), anti-rabbit and anti-mouse IgG secondary antibodies, mouse anti-tubulin monoclonal antibodies and 1400W were purchased from Sigma-Aldrich (Poole, Dorset, England).

### Cell culture and exposure of cells to inflammatory mediators

Normal human fibroblast-like synoviocytes (HFLS) were purchased from Cell Applications Ltd. (Salisbury, England) and cultured in Dulbecco's Modified Eagles Medium (DMEM) containing glutamine (2 mM), penicillin (100 units/ml), streptomycin (100 μg/ml), amphotericin B (0.25 μg/ml) and foetal bovine serum (10% v/v) and incubated in a humidified incubator with 5% CO_2_/95% air at 37°C. Human articular chondrocytes (HAC) were purchased from Cell Applications and cultured in monolayer in chondrocyte growth medium (Cell Applications) as described [20]. Prior to the addition of LPS, HFLS and HAC were seeded overnight in 24-well plates (Greiner; 0.25 × 10^6^ cells/well) then washed once with phosphate-buffered saline (PBS) and media replaced with either serum-free media (*i.e*. unstimulated samples) or serum-free media containing either bacterial LPS (*E. coli* 0127:B8; 10 μg/ml) or a mixture of cytokines (10 ng/ml each of TNF-α and IFN-γ with 1 ng/ml IL-1β) in the presence or absence of GYY4137 (100–500 μM). In some experiments, cells were additionally pre-incubated (1 hr) with either NS-398 (COX-2 inhibitor, 10 μM; [25]), 1400W (iNOS inhibitor, 10 μM; [26]) or PPM-18 (inhibitor of NF-κB activation, 10 μM, [27]) prior to addition of GYY4137/LPS. Thereafter, cells were incubated for a further 24 hrs and the culture medium collected and centrifuged (2 min., full speed) in a microcentrifuge. The resulting cell pellet was processed for COX-2 and iNOS ELISA according to the manufacturer's instructions and Western blotting (TACE, IκBα, phosphor-IκBα) and the aspirate removed for cytokine, NO_2_^−^, IL-6, TNF-α and PGE_2_ analysis [13]. PGE_2_ levels in culture media were determined using a PGE_2_ enzyme immunoassay kit (Cayman, Ann Arbor, MI, USA). TNF-α and IL-6 were assayed by ELISA according to the manufacturer's instructions (R&D Systems) [13]. NO_2_^−^ was determined by Griess assay in cell culture media as described elsewhere [28]. Cell viability assessed after GYY4137 treatment using MTT assay [20] and control experiments showed that concentrations of up to 5 mM GYY4137 did not induce significant cytotoxicity in either HFLS or HAC in this assay. For analysis of the activation of the transcription factor NF-κB using a commercial ELISA (ActiveMotif), the above incubation conditions were repeated using cells seeded overnight in T-75 flasks (3.5 × 10^6^ cells/flask). Nuclear and cytoplasmic fractions were then prepared according to the manufacturer's instructions [9, 13]. Total IκB levels and IκB phosphorylation were determined by Western blotting using antibodies from Cell Signalling Technology.

### Effect of GYY4137 on isolated pro-inflammatory enzymes

To determine whether H_2_S inhibited the catalytic activity of pro-inflammatory enzymes directly, we exposed TNF-α converting enzyme (TACE), COX-2 and iNOS directly to GYY4137. TACE activity was determined using recombinant human TACE enzyme (R&D Systems) and the fluorogenic TACE substrate Mca-Pro-Leu-Ala-Gln-Ala-Val-DPA-Arg-Ser-Ser-Ser-Arg-NH_2_ (R&D Systems). GYY4137 and Na_2_S were prepared at twice the final concentration in assay buffer and the respective H_2_S donor or assay buffer control added to the wells of a black 96-well plate (Greiner Bio-One, Gloucestershire, UK). Recombinant human TACE (30 ng/ml final concentration) was added to each sample well and a recombinant human TACE standard curve (7.5–60 ng/ml final concentration) added to the plate. The plate was incubated for 16 hrs at 20°C. Fluorogenic substrate was then added (7.5 μM final concentration) and the plate protected from light and incubated for 4 hrs at 37°C. Fluorescence was measured on a SpectraMax M2e microplate reader (Molecular Devices, Wokingham, UK) using excitation and emission wavelengths of 320 and 405 nm respectively.

COX-2 activity was determined using the COX Inhibitor Screening Assay Kit (Cayman Chemicals), according to the manufacturer's instructions. GYY4137, Na_2_S and DuP697 (COX-2 inhibitor; Cayman) were prepared in reaction buffer and reaction tubes set up containing recombinant COX-2, haem and either H_2_S donors, DuP697 or reaction buffer only control. Reaction tubes were pre-incubated at 37°C for 1 hr, after which arachidonic acid substrate was added and the tubes incubated for a further 2 min. The reaction was stopped by the addition of HCl and the PGH_2_ produced in the reaction converted to PGF_2α_ by the addition of stannous chloride. PGF_2α_ levels were measured by competitive enzyme immunoassay using a 1:4000 dilution of the samples in EIA buffer. Absorbance at 405 nm was measured on a SpectraMax M2e microplate reader (Molecular Devices).

Nitric oxide synthase activity was determined using the NOS Activity Assay Kit (Caymen Chemicals, Ann Arbor, MI, USA) and L-14C arginine and human recombinant NOS in the presence or absence of GYY4137, Na_2_S or L-N(G) nitroarginine (L-NNA; iNOS inhibitor supplied with the NOS Activity Kit). The reaction tubes were incubated at 37°C for 1 hr, after which the reaction was stopped by the addition of stop buffer. Samples were processed according to the manufacturer's instructions and the amount of radioactive L-citrulline produced measured on a LS 6500 Multi-Purpose Scintillation Counter (Beckman Coulter, High Wycombe, UK).

### Induction of acute joint inflammation in the mouse

Animals were treated in accordance with the Animals (Scientific Procedures) Act 1986 (UK). Male CD1 mice (25–35 g) were used essentially as described previously [29]. Mice were briefly anaesthetized with isofluorane (2%) and injected intra-articular (30-gauge needle) into one rear knee joint (chosen at random) with complete Freund's adjuvant (CFA, 10 μg) in a volume of 10 μl. The other rear knee joint was injected at the same time with an equal volume of pyrogen-free saline as control. Mice were treated with GYY4137 (50 mg/kg, i.p.) or an appropriate volume of vehicle (saline) either 1 hr before or 6 or 18 hrs after CFA injection and all animals were killed at 24 hr. The diameter of both knee joints was measured under isofluorane anaesthesia using calipers (Mitutoyo Inc., Andover, Hamps, UK) both before and at the end of the experiment. The measurements taken were used as an index of knee swelling. All experiments were performed blind in that the investigator was not aware which animals were drug or vehicle injected.

At the end of the experiment, animals were anaesthetized and synovial fluid (∼50 μl in heparinized saline, 5 U/ml) was aspirated from each knee joint. Aliquots of synovial fluid were assayed immediately for H_2_S or stored at −80°C. MPO activity, reflecting the presence of neutrophils, was determined as described previously [4]. The presence of monocytes/macrophages in synovial fluid was assessed using the *N*-acetyl-β-D-glucosaminidase (NAG) assay as described elsewhere [30]. Cytokine (TNF-α, IL-1β, IL-6, IL-8) concentration were determined by ELISA using commercially available kits (R&D Systems) according to the manufacturer's instructions. Synovial fluid H_2_S levels were determined by zinc-trap spectrophotometry [4, 22].

For assessment of drug effects on knee joint structure, tissues from arthritic and non-arthritic control and GYY4137-treated mice were rapidly removed after death, immediately fixed in 10% v/v phosphate-buffered formalin (pH 7.4) for 48 hrs and subsequently embedded in paraffin, fixed as above for 72 hrs and then decalcified in 5% v/v buffered formic acid for 14 days. Sections (4 μm) were cut using a microtome and stained with haematoxylin and eosin. Sections were thereafter examined by light microscopy at either 200× or 600× magnification.

### Statistical analysis

Data are expressed as mean ± SEM with the number of independent observations shown in parenthesis. Multiple comparisons were made by anova followed by *post hoc* Tukey test. Statistical significance of the difference between means was set at *P* < 0.05.

## Results

### Effects of GYY4137 on HFLS and HAC

**Fig. 1:**
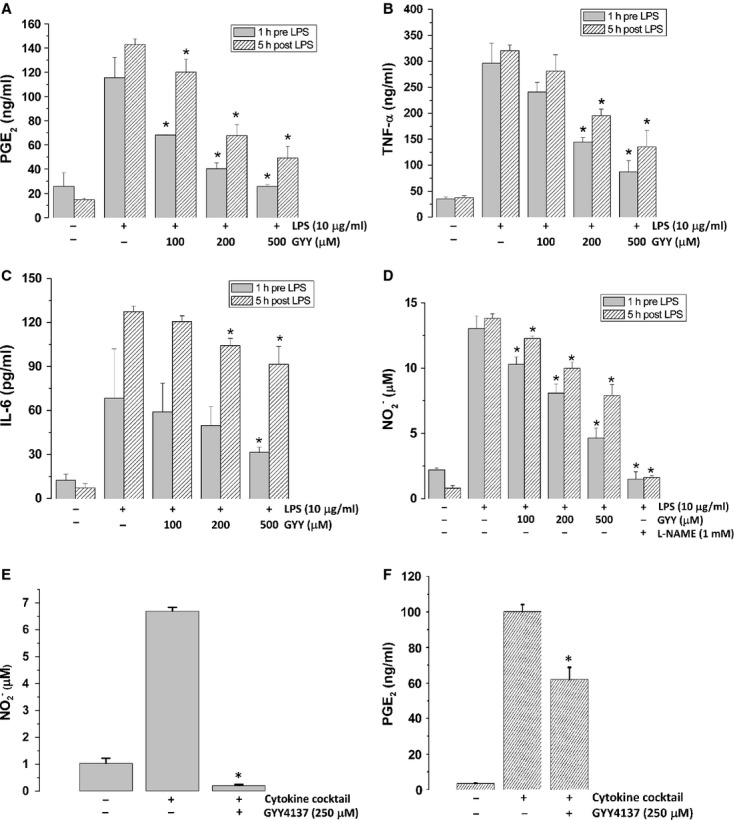
Effect of GYY4137 on LPS- and cytokine-induced synthesis of pro-inflammatory mediators in human synoviocytes (HFLS). HFLS were treated with GYY4137 at the concentrations stated for 1 or 5 hrs post-LPS (10 μg/ml) stimulation for 18 hrs. After this time, cell culture media were collected and analysed for (**A**) PGE_2_, (**B**) TNF-α and (**C**) IL-6 by commercial ELISA and (**D**) ^•^NO (measured as NO_2_^−^) determined by Griess assay. (**E**–**F**) Effect of GYY4137 on cytokine cocktail (comprising of 10 ng/ml TNF-α and IFN-γ and 1 ng/ml, IL-1β)-stimulated NO_2_- (E) and PGE_2_ (F). The iNOS inhibitor (1400W; 100 μM) was added for 1 hr prior to LPS stimulation. Data shown are mean ± SEM of at least three separate determinations. *P < 0.05 *c.f*. LPS/cytokine-stimulated cells.

**Fig. 2:**
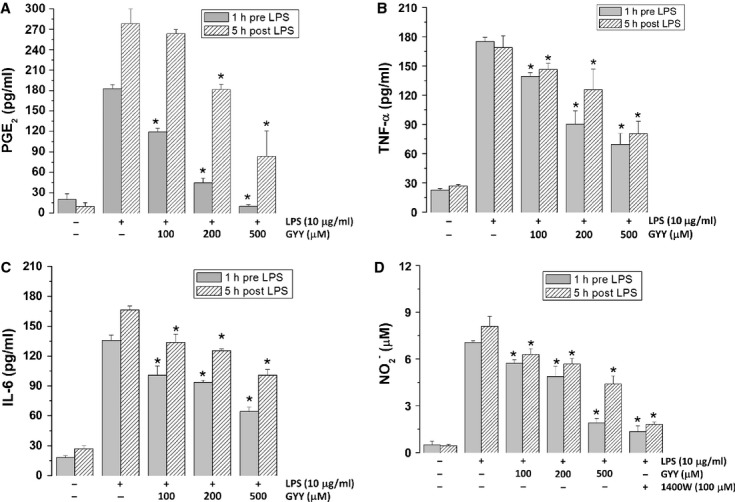
Effect of GYY4137 on LPS- and cytokine-induced synthesis of pro-inflammatory mediators in human articular chondrocytes (HAC). HAC were treated with GYY4137 at the concentrations stated for 1 or 5 hrs post-LPS (10 μg/ml) stimulation for 18 hrs. After this time, cell culture media were collected and analysed for (**A**) PGE_2_, (**B**) TNF-α and (**C**) IL-6 by commercial ELISA and (**D**) ^•^NO (measured as NO_2_^−^) determined by Griess assay. Inhibitors of COX-2 (NS-398; 10 μM) and iNOS (1400W, 100 μM) were added for 1 hr prior to LPS stimulation. Data shown are mean ± SEM of at least three separate experiments. *P < 0.05 *c.f*. LPS-stimulated cells.

Treatment of HFLS (Fig. 1) or HAC (Fig. 2) with LPS significantly increased the levels of PGE_2_ [A], TNF-α [B], IL-6 [C] and ^•^NO (measured as NO_2_^−^) [D] in culture supernatant of both joint cell types. Treatment of HFLS (Fig. 1A–D) or HAC (Fig. 2A–D) with GYY4137 (100–500 μM) for 1 hr prior to stimulation significantly reduced the levels of pro-inflammatory mediators in culture supernatant. Similarly, GYY4137 (>100 μM) significantly inhibited the increase in ^•^NO (Fig. 1E) and PGE_2_ (Fig. 1F) levels induced by a cocktail of TNF-α (10 ng/ml), interleukin-1 beta (IL-1β; 1 ng/ml) and interferon-gamma (IFN-γ; 10 ng/ml).

In preliminary experiments (data not shown), LPS induced an increase in COX-2 and iNOS enzyme levels in a time-dependent manner in both HFLS and HAC with significant increases of each enzyme observed at 5 hrs post-LPS treatment. To determine whether GYY4137 inhibited the activity of these enzymes (*e.g*. PGE_2_ and ^•^NO synthesis) once COX-2 and iNOS levels were induced, cells were stimulated with LPS for 5 hrs, followed by treatment with GYY4137 for a further 18 hrs. GYY4137 significantly reduced the concentration of pro-inflammatory mediators when added to HFLS (Fig. 1) or HAC (Fig. 2) 5 hrs post-LPS-treatment albeit to a lesser extent than selective iNOS inhibitor (1400W).

**Fig. 3:**
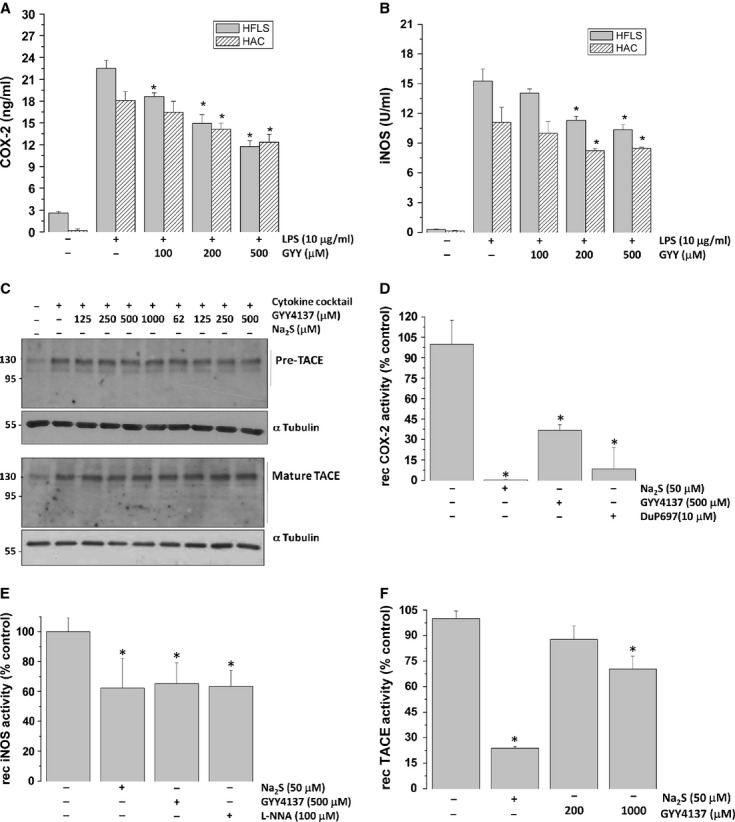
Effect of GYY4137 on LPS-induced expression of COX-2, iNOS and TNF-α-converting enzyme (TACE) expression and activity in human synoviocytes and human articular chondrocytes. HAC and HFLS were treated with GYY4137 at the concentrations stated for 1 hr prior to LPS (10 μg/ml) stimulation for 18 hr. After this time, cells were lysed and levels of COX-2 (**A**) and iNOS (**B**) determined by commercial ELISA. (**C**) Expression of pre- and mature TACE was determined in HFLS by Western blotting after 24 hr. (**D**–**F**) Effects of GYY4137 on isolated human recombinant COX-2 (**D**), iNOS (**E**) and (**F**) TACE activity. Recombinant enzyme activities are expressed as % control enzyme activity after 1 hr (iNOS and COX-2) and 4 hr (TACE). GYY4137 was added at the concentrations stated and enzyme activity determined. DuP697 (1 μM) was used as a positive control for COX-2 activity and L-NNA (supplied with the NOS activity kit; 100 μM) as positive control for iNOS activity. Western blots are representative of three separate determinations and data shown are mean ± SEM of at least three separate experiments. *P < 0.05 *c.f*. LPS-stimulated cells.

To determine whether GYY4137 affected the levels of COX-2 or iNOS enzymes, intracellular levels of these proteins were determined in LPS-treated cells by ELISA. Treatment of HFLS or HAC with GYY4137 for 1 hr prior to LPS significantly reduced LPS-induced COX-2 (Fig. 3A) and iNOS (Fig. 3B) protein levels in addition to reducing PGE_2_ and NO_2_^−^ levels. Although treatment of HFLS with a cocktail of cytokines (TNF-α, IL-1β and IFN-γ) induced an increase in the levels of TACE protein (Fig. 3C), the level of this enzyme was unaffected by GYY4137. To examine further the possibility that the attenuation of LPS-stimulated increases in PGE_2_, ^•^NO and TNF-α (Figs 1 and 2) were due to an inhibitory effect of GYY4137 on COX-2, iNOS and TACE activity, respectively, we incubated human recombinant COX-2, iNOS and TACE with GYY4137 and determined residual catalytic activity. GYY4137 and Na_2_S significantly inhibited COX-2, iNOS and TACE activity (Fig. 3D–F), respectively, suggesting that H_2_S could directly inhibit enzyme activity and consequently cellular synthesis/secretion of PGE_2_, NO_2_^−^ and TNF-α which is independent of any effect of this drug on COX-2 or iNOS protein levels.

**Fig. 4:**
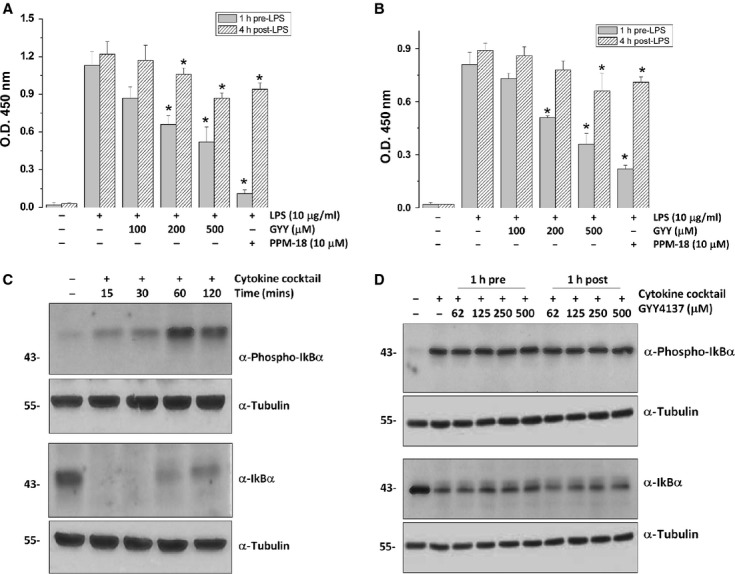
Effect of GYY4137 on LPS-induced NF-κB activation and activity. HFLS (**A**) and HAC (**B**) were treated with GYY4137 or an inhibitor of NF-kB activation (PPM-18) at the concentrations stated for 1 hr before or 4 hrs post-LPS (10 μg/ml) stimulation for 18 hrs. After this time, nuclear extracts were prepared and p65-NF-κB DNA binding determined by commercial ELISA. (**C**) Time course of cytokine cocktail (10 ng/ml each of TNF-α and IFN-γ with 1 ng/ml IL-1β) induced IκBα degradation and phosphor-IκBα phosphorylation in HFLS (**D**) Effects of GYY4137 added 1 hr prior or 1 hr post-cytokine cocktail stimulation on IκBα degradation and phosphor-IκBα phosphorylation in HFLS. Data shown are mean ± SEM of at least three separate experiments. *P < 0.05 *c.f*. LPS-stimulated cells.

We next examined the effects of GYY4137 on NF-κB activation in HFLS and HAC. Treatment of cells with GYY4137 for 1 hr prior to LPS stimulation significantly reduced NF-κB activation in HFLS (Fig. 4A) and HAC (Fig. 4B). Significant inhibition of NF-κB activation was also observed in HFLS (Fig. 4A) and HAC (Fig. 4B) when GYY4137 was added to cells 4 hrs post-LPS treatment although this inhibitory effect was much less marked. This effect of GYY4137 was mimicked by PPM-18 (classical inhibitor of NF-κB activation) although GYY4137 was less effective. Although treatment of HFLS with a cytokine cocktail (containing 10 ng/ml each of TNF-α and IFN-γ with 1 ng/ml IL-1β) for up to 2 hrs resulted in IκBα phosphorylation and degradation of IκBα (Fig. 4C), further Western blotting analysis (Fig. 4D) revealed that GYY4137 did not reduce the cytokine cocktail-induced IκBα degradation or phosphorylation.

### Effects of GYY4137 in a murine model of acute joint inflammation

**Fig. 5:**
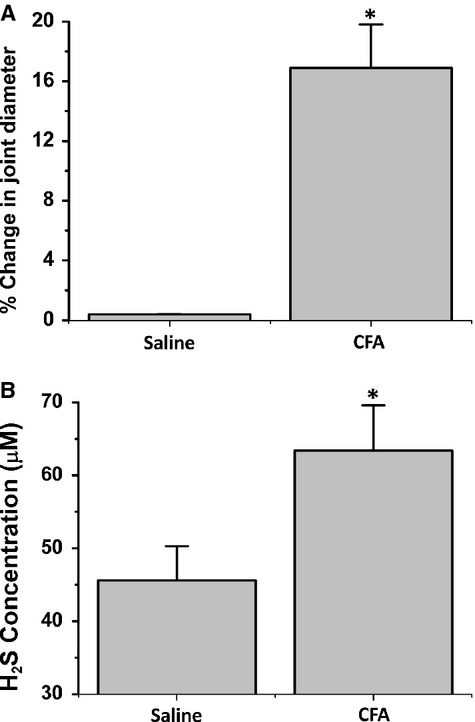
Effects of intra-articular injection of complete Freund's adjuvant (CFA) on knee swelling and synovial fluid levels of H_2_S. CD1 mice were injected with CFA (10 μg in 10 μl) or vehicle (saline) and the effect on knee measured at 24 hr (**A**). (**B**) Effect of CFA treatment on knee joint synovial fluid H_2_S determined by zinc-trap spectrophotometry. Data show mean ± SEM, *n* = 6, *P < 0.05 *c.f*. saline-injected contralateral knee joint.

Intra-articular injection of CFA in the mouse caused significant knee joint swelling measured 24 hrs thereafter. In contrast, intra-articular injection of an equivalent volume of saline into the contralateral knee joint did not cause joint swelling (Fig. 5A). Furthermore, under these experimental conditions, H_2_S concentration was significantly higher in synovial fluid from CFA-injected (*i.e*. swollen) as compared with saline-injected (*i.e*. unswollen), contra lateral knee joints (Fig. 5B).

**Fig. 6:**
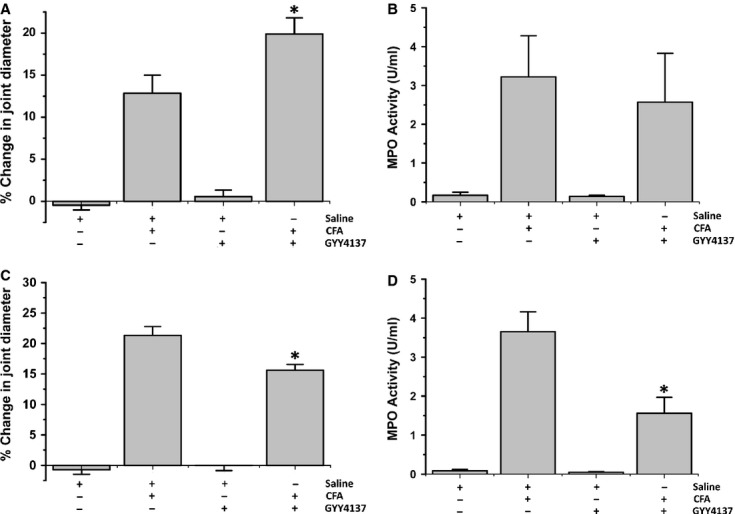
Effects of GYY4137 on knee joint swelling and synovial FLUID MPO activity in CFA-induced acute joint inflammation. (**A**) GYY4137 (50 mg/kg, i.p.) or saline (0.5 ml/kg, i.p.) was administered to mice either 1 hr before (**A** and **B**) or 18 hrs (**C** and **D**) after intra-articular injection of CFA or saline. (A and C) joint swelling. (B and D) Synovial MPO activity. Mice were killed 24 hrs after CFA or saline injection. Data shown mean ± SEM, *n* = 6, *P < 0.05 *c.f*. CFA-injected mice receiving saline.

**Fig. 7:**
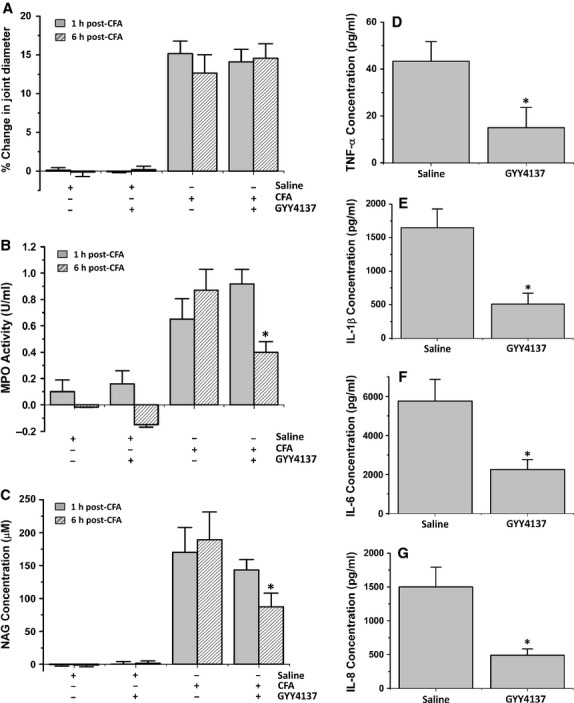
Effects of GYY4137 on CFA-induced joint inflammation. CFA (10 μg in 10 μl) or vehicle (saline) were injected into the knee joints of CD1 mice and GYY4137 (50 mg/kg, i.p.) or saline (0.5 ml/kg, i.p.) administered 1 or 6 hrs thereafter. (**A**) knee joint swelling, (**B**) MPO activity (**C**) NAG concentration measured both 1 and 6 hrs after drug/vehicle injection. Synovial fluid levels of (**D**) TNF-α, (**E**) IL-6, (**F**) IL-1β and (**G**) IL-8 were determined in CFA-injected animals injected 6 hrs after drug/vehicle injection. All mice were killed 24 hrs after CFA or saline injection. Data show mean ± SEM, *n* = 6–8, *P < 0.05 *c.f*. CFA-injected mice receiving saline.

**Fig. 8:**
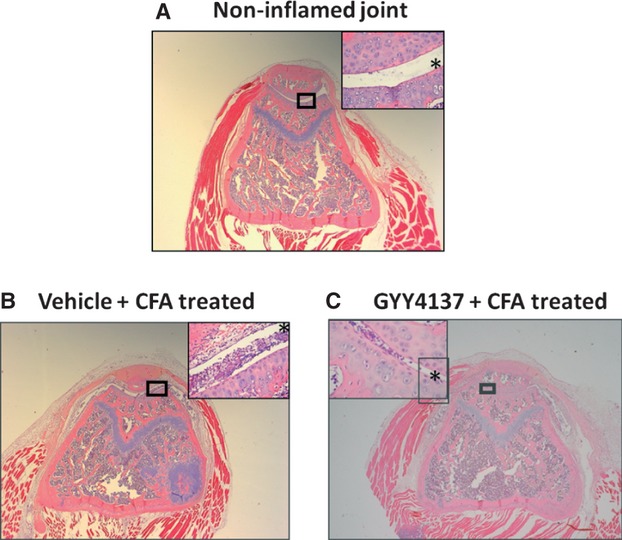
Histological assessment of the effects of GYY4137 on CFA-induced knee joint inflammation. GYY4137 (50 mg/kg, i.p.) or vehicle control saline (0.5 ml/kg, i.p.) was administered to mice 6 hrs after intra-articular injection of CFA or saline and killed 24 hrs thereafter. Figure shows photomicrographs of knee joint sections (200×) and inserts show intra-articular (marked with *) and peri-articular space of knee joints (600×) representative of three separate animals. In these inserts, please note **A**) the absence of inflammatory cells in the intra-articular space of the non-inflamed joint; **B**) the presence of florid inflammation in the same space of a CFA-treated animal; and **C**) the reduction in inflammation achieved in the same space of the GYY4137-CFA-treated animal.

GYY4137 (50 mg/kg, i.p.) or saline was administered to mice either 1 hr before or 6 or 18 hrs after intra-articular injection of CFA. In all cases, animals were killed 24 hrs after CFA injection. Saline did not cause knee joint swelling at any time-point of injection (data not shown). Pre-treatment (1 hr) of animals with GYY4137 resulted in a significant increase in CFA-induced knee joint swelling (Fig. 6A) without any change in synovial fluid MPO activity (Fig. 6B). In contrast, injection of GYY4137 18 hrs after CFA reduced knee joint swelling (Fig. 6C) and also decreased synovial fluid MPO activity (Fig. 6D). Administration of GYY4137 6 hrs after intra-articular injection of CFA did not affect knee joint diameter (Fig. 7A), but did significantly reduce synovial fluid MPO activity (Fig. 7B) and NAG (Fig. 7C), TNF-α (Fig. 7D), IL-1β (Fig. 7E), IL-6 (Fig. 7F) and IL-8 (Fig. 7G) concentrations. Intriguingly, despite the apparent inability of GYY4137 administered 6 hrs after CFA injection to affect knee joint swelling, evidence of reduced neutrophil infiltration and inflammation was also apparent upon histological examination of treated knee joints (Fig. 8).

## Discussion

Hydrogen sulfide has been proposed to be a novel mediator of inflammation. However, the literature is complex and diametrically opposite views on the role(s) of this gas in inflammation have been expressed. We previously proposed that these conflicting observations may be due, at least in part, to the manner in which cells/animals are exposed to H_2_S [1–3, 12]. For example, the vast majority of studies examining the pharmacological effects of H_2_S have used sulfide salts (*e.g*. Na_2_S and NaSH), often at millimolar concentrations, as the source of H_2_S, and have generally concluded that H_2_S is pro-inflammatory (reviewed in [1, 3]). These sulfide salts generate an instantaneous bolus of H_2_S (as well as HS^−^ and Na^+^) which dissipates within seconds whereas endogenous H_2_S synthesis *via* CSE and/or CBS is slow and sustained [12, 13, 24, 31, 32]. As such it is less likely that, *in vivo,* cells would be exposed to a bolus of concentrated H_2_S, as generated with Na_2_S or NaSH (or similar sulfide salts), and more likely that slow-releasing H_2_S donors such as GYY4137 better reflect the role of cell-derived H_2_S in the inflammatory response. Interestingly, unlike sulfide salts, slow-releasing H_2_S donors such as GYY4137 and H_2_S-releasing derivatives of diclofenac or aspirin are generally anti-inflammatory [1–3].

In our current study, GYY4137 caused a range of biochemical effects in cultured synoviocytes (HFLS) and chondrocytes (HAC) *in vitro* which were commensurate with an anti-inflammatory effect of this compound *in vivo*. Thus, GYY4137 produced a concentration-dependent inhibition of ^•^NO (measured as nitrite), PGE_2_, TNF-α and IL-6 production by intact cells. Moreover, in separate experiments, GYY4137 inhibited the catalytic activity of both iNOS and COX-2 *in vitro*. Although Na_2_S has previously been shown to inhibit iNOS activity [33, 34], a direct effect of H_2_S on COX-2 activity has not previously been reported. It seems likely that the molecular target for H_2_S in both enzymes is the haem group [35]. In addition, GYY4137 also reduced iNOS and COX-2 protein levels. NF-κB plays a key part in the transcriptional regulation of both iNOS and COX-2 and is thus a likely target for H_2_S. Inhibition of NF-κB DNA-binding activity by GYY4137 has been observed in isolated rat neutrophils [12], mouse macrophages [13] and *in vivo* in a murine model of endotoxic shock [12]. Moreover, slow-releasing H_2_S donor derivatives of diclofenac [10, 11, 16, 36] and aspirin [37] also inhibit NF-κB activation. Recently, H_2_S-derived either from GYY4137 or from NaSH has been shown to sulfhydrate the p65 subunit of NF-κB at the cysteine^38^ residue, resulting in the formation of a perthiol group on cysteine^38^ and modulation of DNA-binding activity [38]. In this study, GYY4137 did not affect IκBα degradation or IκBα phosphorylation but did inhibit NF-κB DNA-binding activity suggesting this as a likely mechanism of action of this molecule in synovial cells.

An additional novel feature of this study is the finding that GYY4137 reduced TNF-α formation when added to HFLS or HAC either 1 or 6 hrs after LPS stimulation. GYY4137 did not affect intracellular levels of TACE protein or activation in these cells suggesting that the reduction in LPS-evoked synthesis of TNF-α is secondary to inhibition of TACE catalytic activity rather than modulation of TACE enzyme levels. Interestingly, TACE is a zinc-containing metalloproteinase which converts membrane-bound pro-TNF-α to mature and soluble TNF-α [39]. The zinc centres of other proteinases such as angiotension converting enzyme (ACE) are also targeted by H_2_S (albeit from NaSH; >200 μM) and account for the inhibitory effect on ACE activity in human umbilical vein endothelial cells [40]. As H_2_S has a high affinity for zinc, a property widely exploited for the measurement of H_2_S levels and tissue synthesis [2], it is possible that the zinc component of TACE is a target for H_2_S in these cells. Thus, this effect of GYY4137 on TACE activity may account, at least in part, for the inhibition of LPS-induced TNF-α synthesis in HFLS and HAC.

From the foregoing it is obvious that the predominant effect of GYY4137 in cultured HFLS and HAC *in vitro* is anti-inflammatory. Thus, it was of interest to determine whether GYY4137 also reduced inflammation in a mouse model of inflammation.

Complete Freund's adjuvant injection into the knee joint of mice resulted in joint swelling and a significant elevation in synovial fluid H_2_S levels, consistent with a previous study showing increased synovial fluid H_2_S levels in patients with inflammatory joint diseases compared with paired plasma and synovial fluid from osteoarthritis patients [3, 22]. Previous studies in mice have shown increased CSE mRNA levels in the liver and kidney following induction of acute inflammation with LPS, as well as increased CSE enzyme levels in liver following stimulation with TNF-α [4, 38]. Also, *in vitro* studies showed induction of CSE, but not CBS, following stimulation with pro-inflammatory cytokines (TNF-α, IL-6 and IL-1β) or LPS in human articular chondrocytes and peritoneal macrophages [20, 38]. Several studies indicate that the induction of CSE is a key factor towards the increased H_2_S levels observed in acute inflammation. For instance, carrageenan-induced H_2_S synthesizing activity in rat hindpaw was significantly reduced following pre-treatment with the CSE inhibitor PAG [41], as were synovial fluid H_2_S levels [42]. Also, a significant reduction in the TNF-α-induced rise in liver H_2_S levels was observed in CSE^−/−^ mice and peritoneal macrophages derived from CSE ^−/−^ mice [38]. However, the precise roles of enzyme-dependent and enzyme-independent mechanisms of H_2_S synthesis in acute joint inflammation remain to be defined.

Intriguingly, the effect of GYY4137 *in vivo* depended on the timing of its injection. For example, when administered 1 hr before intra-articular CFA (*i.e*. ‘prophylactically’) GYY4137 was not anti-inflammatory but indeed was pro-inflammatory *i.e*. increasing knee joint swelling measured at 24 hr. However, injection of GYY4137 either 6 or 18 hrs after CFA administration (*i.e*. ‘therapeutically’) was anti-inflammatory. These data suggest that GYY4137-derived H_2_S exerts different effects at different stages of the inflammatory response. The pro-inflammatory effect of GYY4137, which is apparent in the early stages of an inflammatory response, is likely due to H_2_S-mediated vasodilatation [12, 24] causing augmented joint blood flow and vascular permeability and thereby exacerbating knee joint swelling. The observation that MPO activity (indicative of the presence of neutrophils) was unchanged in synovial fluid from animals so treated suggests that neutrophils were not involved in the pro-inflammatory effect of GYY4137 when given prophylactically. Interestingly, intra-articular injection of Na_2_S into the mouse knee joint reportedly has the opposite effect *i.e*. synovial microvessel constriction coupled to a fall in synovial blood flow [23]. The reasons for the discrepant data are not clear but differences between the two studies in terms of both the inflammatory agent (carrageenan/kaolin *c.f*. CFA) and the H_2_S donor (Na_2_S *c.f*. GYY4137) used may play a part.

In sharp contrast, GYY4137 administered either 6 or 18 hrs after CFA injection, when knee joint swelling had become established, exhibited anti-inflammatory activity. At 6 hr, synovial fluid MPO activity, NAG (a marker of monocytes/macrophages) and cytokine (TNF-α, IL-1β, IL-6, IL-8) concentrations were reduced by GYY4137 treatment thereby mirroring its effect on synovial cells in culture. GYY4137 has recently been shown to inhibit IL-8 formation *in vitro* [15]. Overall, GYY4137 administered ‘therapeutically’ (*i.e*. after CFA injection into the knee joint) exhibited anti-inflammatory activity most likely by mechanisms which include an effect on leucocyte recruitment and inhibition of pro-inflammatory mediator production.

In summary, we show here that GYY4137, a slow-releasing H_2_S donor, elicits anti-inflammatory activity in LPS- and cytokine-challenged HFLS and HAC as demonstrated by the ability to reduce the levels of pro-inflammatory mediators and cytokines and to ameliorate the induction of pro-inflammatory iNOS and COX-2. In contrast, GYY4137 can exhibit either pro- or anti-inflammatory activity in a mouse model of acute joint inflammation depending on the timing of its administration. Thus, H_2_S affects inflammation in a number of ways including localized vasodilatation and changes in vascular permeability as well as inhibition of mediator release from inflammatory cells. Not only is the anti-inflammatory effect of H_2_S determined by the choice of donor (*i.e*. ‘fast’ *versus* ‘slow’ releasing agent) but also by the timing of donor administration. The anti-inflammatory effects of GYY4137 observed in this study were at concentrations of GYY4137 ranging from 100 to 500 μM. The rate of H_2_S production from this molecule is estimated to be ∼1 μM/hr so much less than 100–500 μM H_2_S was generated. These concentrations of GYY4137 had no effect on cell viability under the conditions used in this study (data not shown), but could potentially introduce side effects in therapeutic applications. Therefore, both the timing and effective concentration of H_2_S donor should be borne in mind when considering the development of novel anti-inflammatory agents based on the principle of H_2_S donation.
